# Gender perspectives in resilience, vulnerability and adaptation to global environmental change

**DOI:** 10.1007/s13280-016-0842-1

**Published:** 2016-11-22

**Authors:** Federica Ravera, Irene Iniesta-Arandia, Berta Martín-López, Unai Pascual, Purabi Bose

**Affiliations:** 1 ICAAM - Instituto de Ciências Agrárias e Ambientais Mediterrânicas, LDSP - Landscape Dynamics and Social Process Research Group, Universidade de Évora, Pólo da Mitra, Ap. 94, 7002-554 Évora, Portugal; 2 CREAF, Cerdanyola del Vallès, 08193 Catalonia, Spain; 3 Social-Ecological Systems Laboratory, Department of Ecology, Universidad Autónoma de Madrid (UAM), Calle Darwin nº2, Campus de Cantoblanco, Madrid, Spain; 4 Faculty of Sustainability, Institute of Ethics and Transdisciplinary Sustainability Research, Leuphana University of Lüneburg, Scharnhorststr. 1, 21335 Lüneburg, Germany; 5 Basque Centre for Climate Change (BC3), Edificio Sede Nº 1, Planta 1ª, Parque Científico de UPV/EHU, Barrio Sarriena, 48940 Leioa, Spain; 6 IKERBASQUE, Basque Science Foundation for Science, Maria Diaz de Haro 3, 6 floor, 48013 Bilbao, Spain; 7 Department of Land Economy, University of Cambridge, 19 Silver St., Cambridge, CB3 9EP UK; 8 Consultant within Indigenous Peoples team of Food and Agriculture Organisation (FAO), 416 Sai Section, Ambernath, Mumbai, 421501 India

**Keywords:** Climate change, Feminist environmentalism, Gender research, Intersectionality, Sustainability

## Abstract

The main goal of this special issue is to offer a room for interdisciplinary and engaged research in global environmental change (GEC), where gender plays a key role in building resilience and adaptation pathways. In this editorial paper, we explain the background setting, key questions and core approaches of gender and feminist research in vulnerability, resilience and adaptation to GEC. Highlighting the interlinkages between gender and GEC, we introduce the main contributions of the collection of 11 papers in this special issue. Nine empirical papers from around the globe allow to understand how gendered diversity in knowledge, institutions and everyday practices matters in producing barriers and options for achieving resilience and adaptive capacity in societies. Additionally, two papers contribute to the theoretical debate through a systematic review and an insight on the relevance of intersectional framings within GEC research and development programming.

## Prologue

**Fig. 1:**
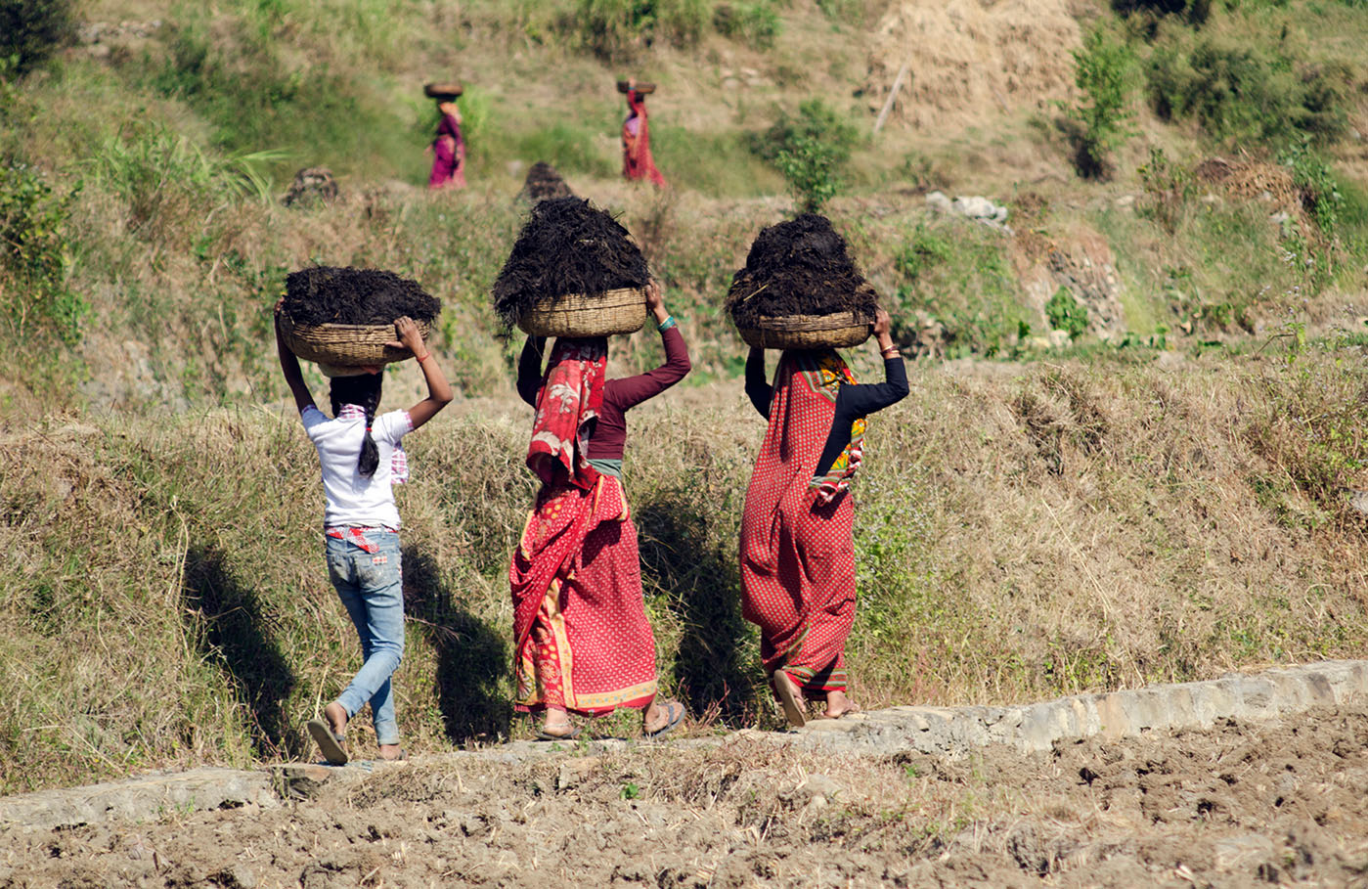
From mother to young daughter building strategies to adapt to change. When the young girl in jeans and white T-shirt comes back from school in the afternoon, she joins the women of the village in carrying heavy baskets of cow dung to return soil fertility to the fields. Sixty-five percent of the mid-high Kumaon hills are forest covered, and farming is largely done on the rain-fed uplands whose soils are protected from erosion by terracing. In this sensitive landscape, the women’s collaborative goal is to minimize the vulnerability of local livelihoods to environmental change. Reproduction precedes social production. There has been a clear recognition that “gender” is relevant in community agroforestry. Managing soil fertility and forest resources, and conserving seed and knowledge exchanges are mainly women’s roles. They are assured by collaborative safety nets, ninety percent of which are run by women. These socio-cultural strategies, transmitted from mothers to young women, underlie individual and collective capacity to adapt to crisis and long-lasting change (Ravera and Tarrasón 2014). (Photo: D.Tarrasón)

**Fig. 2:**
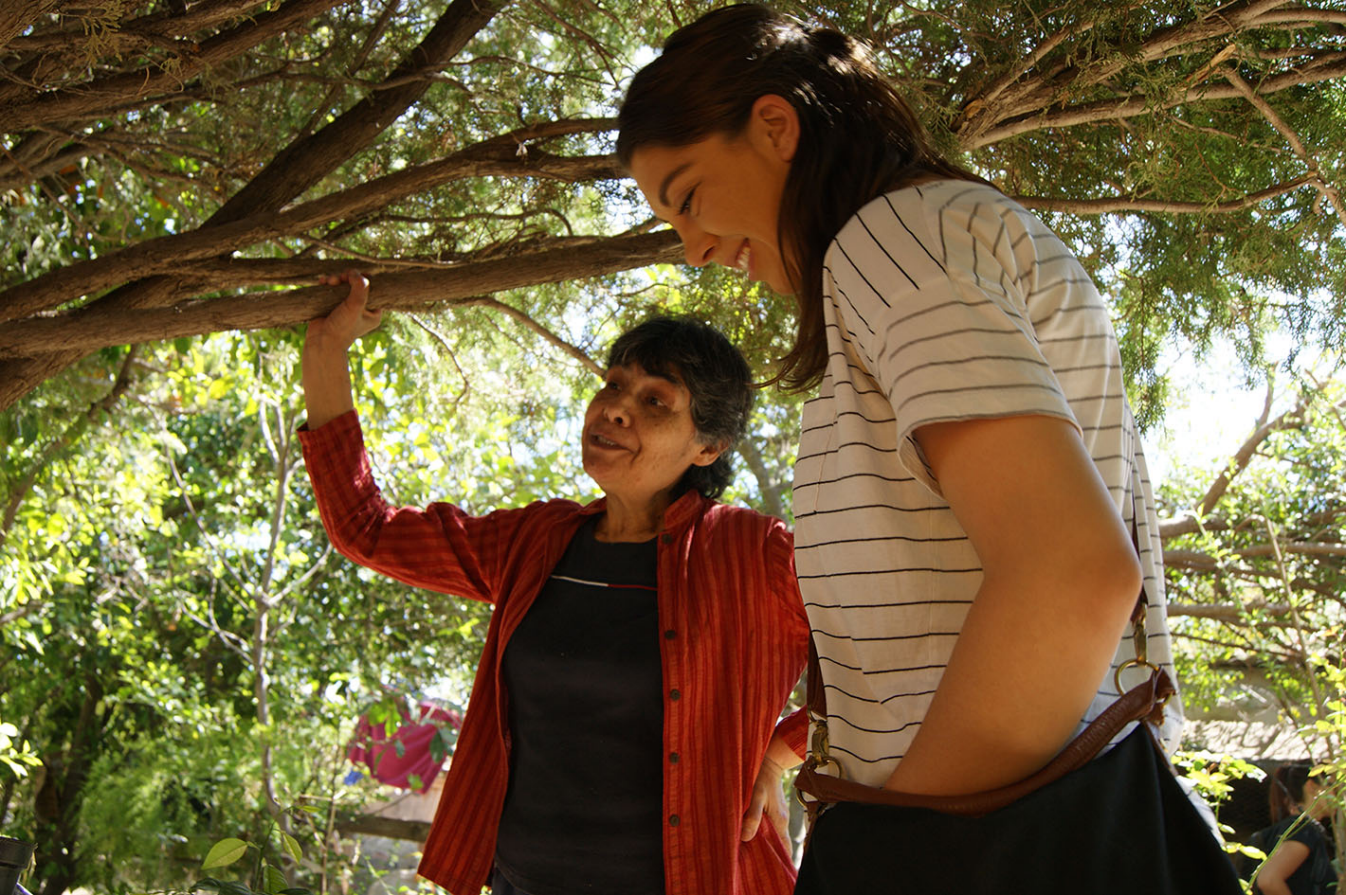
Angela’s home garden for innovative adaptation. Women’s agricultural production in home gardens in Sonora, Mexico is invisible to policymakers, thus programmes and policies are not developed to support and/or build on women’s climate and water-related adaptation strategies. In Angela’s small orchard in her home garden she makes use of grey water from her washing machine for irrigation to save water and maintain healthy fruit trees despite higher temperatures. (Photo: S. Buechler)

Figures 1 and 2 tell us two stories collected in this special issue, centred on acknowledging the complex connection between gender and global environmental change. Across all continents, cultures, races and ethnic groups, women generally experience negative impacts due to environmental change. Despite their vulnerability, women are also increasingly proactive in negotiating and adopting individual and collective innovative strategies for dealing with and adapting to environmental change. Thus, we can ask ourselves whether *gendered and alternative knowledge and everyday work are relevant in global environmental change.*

Though gender is recognized as a significant dimension of environmental change, sustainability and development (e.g. Agarwal 2010; Arora-Jonsson 2014; Carr and Thompson 2014; Leach 2016), gender analysis of socio-environmental issues still remains understudied, and its incorporation in development and environmental policies has advanced little by little. To contribute in filling such gaps has been the impetus of this special issue. Through the papers of this collection, we adopt a gender lens to unpack complex issues of differential sensitivity and adaptive capacity of individuals and societies to global environmental change (hereafter GEC). Indeed, the contributions not only analyse how future resilience, adaptation and mitigation of GEC are largely shaped by roles, responsibilities and entitlements associated with various markers of social identities and power relations, including gender, but also ethnicity, socio-economic class or caste (e.g. Adger 2006; Reid and Vogel 2006; Segnestam 2009; Djoudi and Brockhaus 2011; Carr and Thompson 2014). The intersection of these social factor constraints also provides differential opportunities to individuals and societies for adaptation and transformation in a resilient way in the face of GEC.

The main goal of this special issue is thus to offer a room for an interdisciplinary and engaged research in GEC, where gender plays a critical role in building resilience. The collection of papers aims to trace the lineage of arguments which link gender and diversity of identities within GEC, presenting how they recur in new forms of resilience, vulnerability and adaptation studies.

In the following section, we describe the setting and key questions that have inspired the special issue. The second section introduces the core concepts and approaches of vulnerability, resilience and adaptation to GEC as well as the explicit focus adopted through our gender lens. By addressing the linkages between gender and feminist studies and GEC research, we move progressively away from a gender mainstreaming focus on women’s vulnerabilities and we embrace a wider focus necessary to analyse the significance of the active roles of women in their efforts to adapt to and mitigate effects of GEC through their collaborative actions, situated knowledge^1^ and embodied practices. In the last section, we summarize the papers which are part of this collection; one literature review of the interlinked topics of gender and climate change, nine empirical papers and a final insight paper. The special issue closes with an insight paper that synthesizes the main theoretical contributions and methodological advances from gender studies in GEC research and comes up with a suggested agenda for the next years to adequately inform effective environmental and development policies at different organizational levels—from local to global.

## Building the dialogue between scientific fields: Why to bring gender in global environmental research

Human activities have been emerging as a main force shaping the biosphere from local to global scales (Rockström et al. 2009). The studies included in this special issue well illustrate some of the recent changes in political, institutional, economic and environmental issues that have had severe consequences for natural and human systems across continents, threatening the capacity of social–ecological systems to recover from shocks and to maintain key functions that are able to preserve the resilience in the system in every specific rooted location (Biggs et al. 2015).

This is a time of unprecedented challenges for achieving a sound transition towards sustainability, in which overlooking poverty and inequality (including gender inequality) constitutes a major threat to human well-being and the ecological functioning of the planet. Indeed, as the United Nations’ 2030 Agenda on Sustainable Development Goals (SDGs) highlight, current sustainability challenges are about equality and social justice as much as about biodiversity, ecosystems and the environment and they need to be addressed as an “indivisible whole” (Nilsson et al. 2016).

In this context, social–ecological research warns about “the need to seek alternative forms of thinking about, and action toward, the world around us” (Ogden et al. 2013, p. 346). However, while research on socio-ecological resilience mostly focuses on the severe consequences of GEC for natural and human systems, development studies address the impact of human development on socio-economic inequalities, without a clear emphasis on the distribution of differential ecologically based consequences on “everyone who plays a role in a social–ecological system” (Nelson and Stathers 2009, p. 65).

To embrace such intertwined interactions between resilience and development studies to respond to sustainability and equity challenges, the research community as well as decision makers need to engage with the diversity of voices and forms of knowledge of multiple social agents. Additionally, they need to bridge diverse theories, approaches and disciplines that can generate a constructive debate, and eventually lead to more suitable solutions in the face of unprecedented global (including environmental) changes.

A recognition of the significant dimension of gender in environmental sustainability and development is quite recent (UN Women 2014; Verma et al. 2014; Leach 2016), but overall the gender perspective in GEC continues to be underexplored by the scientific community (Banerjee and Bell 2007; MacGregor 2009)^2^ and the incorporation of such perspective into development programmes and environmental policies has been, to date, insufficient and fragmented (Agarwal 2010; Arora-Jonsson 2014; Carr and Thompson 2014). This general lack of attention to gender issues in GEC research might be associated with a very technical approach to the GEC debate, a late incorporation of the social sciences and with the low representation of women in global science-policy initiatives that act as catalysts for co-producing scientific and policy-relevant knowledge, such as within the Intergovernmental Panel on Climate Change (IPCC) and the Intergovernmental Platform on Biodiversity and Ecosystem Services (IPBES) (Corbera et al. 2015; Montana and Borie 2016). Although, representation is an important issue it should be highlighted that by itself a good representation of women in policy-making bodies does not assure that attention to gender aspects will be increased (Kaijser and Kronsell 2014).

To date, GEC research has mainly focused on women as unitary subjects, opposed to men. Surprisingly a very small amount of the literature focuses on the complex “intersectional character of gender and power relations” issue in building resilience and adaptive (or maladaptive) capacities (Harris 2006; Carr 2008; Arora-Jonsson 2011; Tschakert and Machado 2012). Additionally, there is a tendency to frame the discussion on women being especially vulnerable to broader environmental and structural forces, rather than focus on women and other marginalized groups being active agents for transforming and adapting to change, collectively and from the margins (e.g. Agarwal 1992; Mies and Shiva 1993; Resurrección 2013; Meinzen-Dick et al. 2014; Buechler and Hanson 2015).

The main attempts to fill this research lacuna have been conducted from a feminist environmentalism, geography and political ecology perspectives (e.g. Harris 2006; Nightingale 2006; Resurreccion and Elmhirst 2008; Agarwal 2010; Elmhirst 2011; Nightingale 2011; Buechler and Hanson 2015). This special issue further contributes to such initial contributions as it aims to illustrate new directions of interdisciplinary research at the intersection between (up to now) not sufficiently well connected research fields and action. Particularly, three key questions inspire the special issue:

- Why is it relevant and necessary to address a more nuanced gender and feminist perspective in GEC studies?
- How and to what extent are the issues of gender and diversity of identities connected to resilience and adaptation?
- How can the scientific community bridge theoretically and methodologically the studies on gender and resilience, vulnerability and adaptation to contribute to transformational future pathways?

A key step in a self-reflective interdisciplinary dialogue between resilience and gender and feminist studies took place in the Resilience 2014 Conference in Montpellier (France),^3^ where scholars from several research communities as well as local practitioners explored and attempted to bridge the concepts of adaptation, social vulnerability, transformation and development (Bousquet et al. 2016). Focusing on resilience thinking, the conference offered the opportunity to explore how to articulate multiple paradigms, concepts and methodologies that belong from different disciplines. Despite the initial interest in the social dimensions of resilience, surprisingly little focus was given to gender perspectives and only one session within the Conference targeted the topic under the general theme of “social sciences perspectives on resilience”. It was in the discussion of such session where attendees critically reflected on the lack of attention to key gender issues in the research of sustainability, resilience, vulnerability and adaptation.

From that initial conversation among gender and feminist scholars, sustainability scientists and development researchers and practitioners, the idea of this special issue was seeded. Linking theories and conceptual frames among, to date, separate research fields are illustrated in this special issue through empirical studies around the world, such as in Oceania, Central and South Asia, Africa, South and North America and Europe. In selecting the studies, the purpose is to engage a broad environmental audience who are not used to apply any specific gender and feminist lens. Although the studies presented in this special issue apply different frameworks for bringing gender into GEC research, they all present some commonality in addressing gender not as uncontested analytical term, but rather as a complex view which disentangle power dynamics and as a multidimensional perspective which intersects with other dimensions of social differences such as caste, social status and class, education, ethnicity, etc.

## Linking disciplines, concepts and frameworks

### Resilience and adaptation in global environmental change research

Resilience, vulnerability, adaptation and transformation, are central concepts in framing our analyses of GEC and the challenges of sustainability (Miller et al. 2010; Turner 2010). Despite the potential linkages between vulnerability and resilience frameworks, there remains some division between the two frameworks mostly due to conceptual constructs, scientific traditions and lack of interaction between the two academic communities involved (Miller et al. 2010; Turner 2010; Engle 2011; Turner 2013).

On the one hand, the concept of vulnerability is rooted in hazard and disaster risk reduction studies as well as in development studies on food security, poverty and sustainable livelihoods (Miller et al. 2010). Vulnerability approaches are actor-oriented approaches with their concurrent emphasis on values, interests, agency and knowledges (for a revision see Adger 2006).

On the other hand, the resilience perspective originates from complex system thinking, with a strong natural science influence (Miller et al. 2010; Turner 2013). Some of its most innovative aspects have been the fundamental role of adaptive capacity, the importance of internal change in shaping social–ecological systems and its holistic approach that embraces complexity (Cote and Nightingale 2012; Tschakert and Tuana 2013). Some authors (Folke et al. 2010; Cretney 2014) have also highlighted as important features of resilience thinking the concept of transformability or the ability to create “a fundamentally new system when the ecological, economic, or social structures make the existing system untenable” (Walker et al. 2004).

One of the major points of contention between the two frameworks has been the treatment of agency and power, seen as a core strength of the vulnerability approach but insufficiently tackled in resilience approaches (Cote and Nightingale 2012; Turner 2013; Fabinyi et al. 2014). Critiques have argued that normative aspects such as power relations and cultural values, seen as essential to the development and functioning of social–ecological systems, have been underexplored resilience approaches, reflecting an overemphasis on biophysical shocks and disturbances to the detriment of social and political change (Cote and Nightingale 2012; Turner 2013; Fabinyi et al. 2014).

However, given the urgency of global environmental and social change challenges, in recent researches a shift from fragmented studies towards a more complex and comprehensive perspective that brings together system dynamics understanding and regime shift studies with questions of agency, power and equity in addressing socio–ecological systems changes and sustainability has been advocated (McLaughlin and Dietz 2008; Miller et al. 2010; Tschakert and Dietrich 2010). In this context, certain confluence between resilience and vulnerability approaches seems to be taking place (Brown 2014; Biermann et al. 2015).

Specifically, answering to Cote and Nightingale’s (2012) call to embed the concept of resilience in social relations of power, knowledge and culture, Tschakert and Tuana (2013) propose reframing both the resilience and vulnerability frameworks based on the work of feminist theorists such as Judith Butler and Donna Haraway. They caution against a deceptive reconciliation between vulnerability and resilience approaches that does not overcome problematic dichotomies that have constrained both frameworks. One of them is the understanding of vulnerability as resilience’s opposite where vulnerability is seen as bad and to be reduced and resilience as good and to be enhanced. From the relational perspective they propose, vulnerability is, thus, not framed as passive or negative where to be vulnerable is to be susceptible, exposed or at risk. Rather, vulnerability is conceptualized as an ability to affect and be affected and it is rather the condition of being in relation to others. Resilience is always partial and positioned, always for a particular collection of entities in a particular context, always power sensitive and emergent from webs of relationships. Therefore, the goal is not fostering invulnerability but finding better ways of encouraging relations between peoples, current and future, and between peoples and places.

In this sense, contributions from gender and feminist studies, as we shall see in this special issue, can help to develop these notions of situated resilience that authors have been proposing.

### From gender mainstreaming to fragmented identities

In the last decades, there have been many calls that claim for integrating gender perspectives into sustainability discourses (e.g. Agarwal 2010; UN Women 2014; Leach 2016). Different international agreements, such as the Rio Declaration on Environment and Development (UNCED 1992) or the United Nations document on Sustainable Development (UN 2012), recognize the important role of gender equality in decision making for achieving sustainability goals (UN Women 2014). Gender and identity are critical aspects for analysing GEC, as van Dijk and Bose (2016, p. 14) explain: “gender is the culturally defined roles of men and women and more generally identity—which one pre-supposes specific roles in social and political life—indicate who one is and determines to a large extent what kind of roles one can take, the activities one can undertake and the kind of rights one has with respect to (environmental) resources”.

Since the mid-1970s, the interest of gender in environmental research has been increasingly developed by different scholars and social organizations [i.e. ecofeminism, Women, Environment and Development (WED) or Gender and Development (GAD)] (Bhavnani et al. 2003; Arora-Jonsson 2014; Meinzen-Dick et al. 2014; Leach 2016). Ecofeminism, the term first used by Françoise d’Eaubonne in 1974, refers to feminist issues with ecological concerns, emerging as an outcome of male oppression. Early ecofeminist scholars pointed out that women are more connected and closely linked with nature than men (e.g. Shiva 1988; Mies and Shiva 1993). The WED debate is closely linked to the impact environmental development had on women (Shiva 1988). It takes a critical view of development policies wherein it explores the interface between technology and modernisation vis-à-vis environmental changes. Further, the WED approach often conceptualized women as victims of environmental degradation and linked the degradation of nature with the oppression of women through patriarchy (Meinzen-Dick et al. 2014; Leach 2016). In all of these approaches, women were viewed as a static, uniform and homogeneous group; aspect that was criticized in the 1980s and 1990s by new strains of research in gender and environment (Sandilands 1999; Gaard 2011). For example, the so-called ‘feminist environmentalism’ aimed to understand the women’s and men’s relationship with the environment by considering the interactions between gender and class, different ecological dimensions and the effects of environmental change (Agarwal 1992; Seager 2003). The GAD approach was an ideological shift ‘from women to gender’ in a way emerged from the realization that unlike WED that advocates women’s participation there is a need for gender-responsive planning and policy-making that recognizes gender equity in environment management.

In the middle of the 1990s, feminist political ecology introduced a conceptual framework for examining human–environmental issues, explicitly addressing critical issues of power, giving also emphasis to gendered forms of knowledge and gendered governance structures of the environment (Rocheleau et al. 1996; Nightingale 2006). More recently, feminist political ecology has incorporated new theoretical approaches to understand how different gender identities, associated with other identities, are co-produced through power relations, shaped in everyday life, in a dynamic and negotiation space, explaining different interactions with land, water, trees or other natural resources (Elmhirst 2011; Nightingale 2011). The ‘intersectional’ approach can be found among these theoretical approaches, first coined in the 1990s by Crenshaw (1991) and long present in black feminist thought, that aims to understand how different axes of experience and identity (e.g. gender, sexuality, class, caste, race, age, education, access rights) intersect and produce different effects that could not be explained by analysing single categories (Nightingale 2011). Gender is linked with a sense of belonging with respect to ethnicity, place of origin, language or religion. The politics of social identity in gender is thus closely related to inclusion and/or exclusion to rights of access, use and management of forest, land, water and other natural resources and their unequal distribution within households and communities, strictly related to cultural significance and a sense of belonging (Bose 2012).

In spite of these advances in the literature regarding gender and environment, very small amount of this literature focuses on the complex “intersectional character of gender and power relations” in building resilience and adaptive capacities to GEC (Harris 2006; Arora-Jonsson 2011; Tschakert and Machado 2012). Additionally, some scholars have warned against the return of renewed WED discourses in climate change agendas (Resurrección 2013). Additionally, the new theoretical debate within feminist and gender literature has created a vibrant opportunity for seeking interdisciplinary efforts and methodological advances as a way of expressing the multiple voices and change policy outcomes (Rocheleau 1995; Nightingale 2003; Kaijser and Kronsell 2014; Nightingale 2016). Therefore, the present special issue aims to fill this knowledge gap by addressing theoretical and methodological challenges in gender research of resilience and GEC.

## Major themes and paper contributions

Two main specific objectives are addressed through the papers in this special issue: (1) to frame and advance the research on gender, resilience and adaptation to GEC in a comprehensive and interdisciplinary way and (2) to apply such interdisciplinary approach in a number of empirical cases from countries in the Global South and the Global North, where the link between the fields of resilience, vulnerability, adaptation and gender is innovatively developed.

The first objective aims to contribute to the theoretical debate on the link between resilience, vulnerability and adaptation research, and feminist studies. The special issue thus provides key insights for designing and operationalizing development interventions and policies that tackle GEC across scales. Two papers (Djoudi et al. 2016; Thompson-Hall et al. 2016) open and close the special issue, contributing to such theoretical debate.

Djoudi et al. (2016) conduct a review in order to determine whether gender is framed from the intersectional approach in climate change adaptation. They show that intersectionality is not sufficiently considered by this body of knowledge and that gender is basically approached from a simplistic perspective of men-versus-women. In addition, their paper argues that this oversimplified inclusion of gender perspectives can lead to misconceptions about gendered aspects of vulnerability and, thus, can result in misguided recommendations towards building adaptive strategies and increasing socio-ecological resilience in the face of global change and environmental stressors, such as in the case of climate change.

The special issue ends with another theoretical reflection by Thompson-Hall et al. (2016) on intersectional framings within research and development programming. After presenting the evolution of feminist studies, the authors argue how intersectionality from political ecology and feminist geography gives deeper attention to multiple facets of farmer identities in rural contexts, understanding dynamic assemblages of power and institutions and how these assemblages shape sensitivity and adaptive capacity of different people. According to the authors, engaging intersectionality in agrarian settings applicable to issues that link climate change, livelihood strategies and agroecosystem management, implies the need for orchestrating a diversity of disciplines and empirical tools, such as participatory and feminist geographic information system (GIS) or ecosystem service approaches. The authors show that such orchestration, for instance, aims to build a gender-focused research on climate-smart agriculture, while at the same time to develop more holistic understanding of vulnerability and adaptive capacity of farmers across agrarian landscapes.

**Fig. 3:**
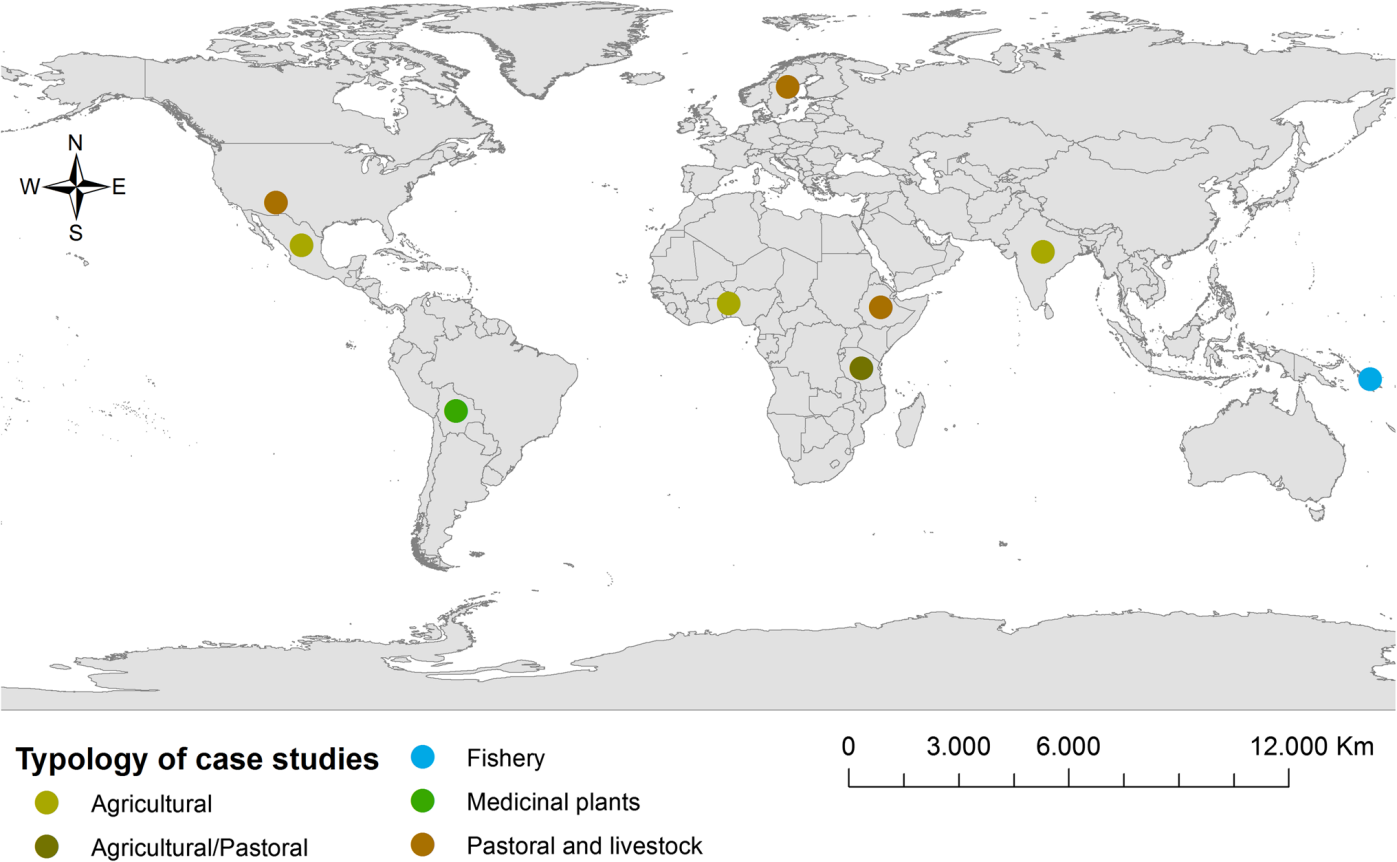
Map of case studies showing the diversity of agro-environmental settings related to agricultural management, pastoral and livestock management, medicinal plants and fishery in this special issue

The second objective of this special issue is to have an overview of possible cases that through place-based research are able to link gender and vulnerability, resilience and adaptation to GEC. The empirical cases selected for the special issue come mainly from countries in the Global South and also from the Global North (see Fig. 3), where this link is rather unexplored. Such place-based approach is probably the best way to understand how gendered diversity in knowledge, institutions and everyday practices matters in producing barriers and options for achieving resilience and adaptive capacity of societies. Additionally, the comparison among empirical cases allows inquiring the context-specific processes of renegotiation of gender relationships and livelihoods, under multiple dynamics and in response to environmental change and other stressors.

All authors contributing to the special issue address the complex intertwining components of social–ecological systems and the ways women are individually and collectively affected by GEC as well as how they differently attempt to respond to GEC. The rich texture of the case studies helps to show the importance of contextualizing the debate within different socio-cultural situations to identify effective and equitable decision-making options that would respond to the adverse effects of GEC. In the line of recent critical literature, the set of empirical studies in this special issue questions obvious and simplistic arguments of the predominant research on gender and GEC, thus going beyond unrealistic notions of gender-specific impacts where women are often claimed as automatically and immediately more disadvantaged and vulnerable than men (especially in the Global South) or gender-specific responses where women are generally portrayed as more environmental conscious than men (especially in the Global North) (Arora-Jonsson 2011; Elmhirst 2011; Tschakert 2012). Indeed, the papers of the special issue apply a feminist theoretical lens that shift away from such dichotomies men/women, virtuous/vicious and South/North, challenge a masculine technical and expert knowledge in GEC and unpack power axes and fragmented identities that differently shape situated (i.e. local and context specific) sensitivities and adaptive responses.

Three main themes cluster the empirical cases bridging gender and GEC: (1) the development and application of local ecological knowledge in historically and geographically, cultural and social context-specific situations, (2) the enhancement of governance structures and (3) the everyday practices that are able to respond and adapt to global change.

### Towards a situated knowledge on resilience and adaptation

Resilience scholars consider that bodies of cultural knowledge in relation to the environment (referred most commonly as folk knowledge, indigenous knowledge, traditional ecological knowledge, local ecological knowledge or local environmental knowledge) constitute a key analytical domain of social–ecological systems research (Berkes et al. 1998; Folke 2006). Under such broad and complex view, local ecological knowledge is thus recognized as a hybrid, dynamic and adaptive result of differentiated synthesis and evaluation processes (Nygren 1999), rather than a unitary system, static and in decline. While local ecological knowledge retains the cultural and place-based characteristics (Ellen et al. 2000), its maintenance is highly threatened by GEC (Pardo-de-Santayana et al. 2010; Hernández-Morcillo et al. 2014). As crucial middle ground between science and policy, resilience and adaptation approaches open up space for including lay and indigenous knowledge in research as an object of study and also as a tool for scientific inquiry of the human dimensions of environmental change (Fairhead and Leach 1996; Fortmann 2008). From a critical social science perspective, scholars criticize the need for including normative questions in resilience research with respect to knowledge production (Cote and Nightingale 2012). They propose to look at the “situated knowledge” which is mediated in every historical and geographical context by relations of power between socially differentiated groups and their cultural representations. Under this topic, two papers (Díaz-Reviriego et al. 2016; Smucker and Wangui 2016) reflect from different perspectives and methodological approaches on the role of local ecological knowledge in building resilience and the multidimensional aspects related to knowledge production in shaping adaptation in the face of current social and ecological dynamics.

Díaz-Reviriego et al. (2016) apply resilience theories to a case study amongst lay Tsimane’ of Bolivian Amazonia and their local ecological knowledge system related to the elaboration of the “rules in use” of ailments. Drawn from ecological concepts, the paper introduces and tests the concepts of knowledge, diversity and “functional knowledge redundancy” in the assessment of the degree to which local medicinal knowledge systems may be adaptive and resilient to GEC that are affecting local medical systems and community resilience. Through a mixed method analysis, the paper reflects on knowledge distribution, and specifically gendered knowledge, within a local medical system. It finally discusses how differences in access to resources, division of labour and caregiving tasks define gender relations and gendered content of lay knowledge and preferences, finally affecting well-being and adaptive capacity of Tsimane’ communities.

Rather than focus narrowly on “traditional” forms of knowledge, Smucker and Wangui (2016) present differentiated and dynamic local knowledge that shape the contemporary set of adaptive practices identified as most effective in reducing local climate risk in two communities of the Kilimanjaro Region in northern Tanzania. Shifting the attention from the content to the context of knowledge production to cope with climate and other stressors, the paper reflects on agency and power at the margins. The authors argue that while established forms of culturally based knowledge can inform the management of complex agro-ecologies, women’s strategies are able to build social networks, access resources and gain access to formal institutions. As conclusion, authors suggest that knowledge production at the margins raises important questions about the prospects for expanding the knowledge domain to address access mechanisms to resources and for integrating knowledge systems (i.e. local and external or traditional and scientific) to guide planned adaptation.

### Gendered institutions and global environmental change governance

Social–ecological resilience is highly dependent on both ecological and social dynamics and in turn both are determined by the governance and institutional context (i.e. informal and formal norms and rules) that shape the co-evolution of social–ecological systems over time (Biggs et al. 2015). Such institutional context also determines gendered roles with regards to access, control and use of natural resources (Rocheleau and Edmunds 1997; O’Shaughnessy and Krogman 2011). Hence, understanding the extent to which institutions, especially informal ones, are gendered (e.g. customary norms over communal property rights may discriminate against women’s needs or intra-household gendered relations may favour men’s preferences over land use) is a critical aspect in our goal for understanding adaptation and resilience to environmental change through a gendered lens. The special issue recognizes this aspect by including three papers (Aregu et al. 2016; Cohen et al. 2016; Dah-gbeto and Villamor 2016).

Aregu et al. (2016) analyse the gendered roles in the management of communal pastures in the highlands of Ethiopia and the consequent effects on the resilience of this traditional social–ecological system. They find that the informal institution regarding the customary norms and rules for accessing the communal pastures discriminates against women. They point out that such discrimination of women has direct implications for the resilience of the communal pasture system as it overlooks women’s knowledge regarding future adaptation options and threatens to undermine the legitimacy of the management system. Through a qualitative study, the authors conclude that such gender blindness is highly problematic as the exclusion of women’s needs, views and knowledge from the management of communal pastures undermines the community’s capacity to adapt and cope with critical social–ecological challenges; spread of a poor quality grass and the perpetuation of socio-economic inequality.

Dah-gbeto and Villamor (2016) shed light on gender differentiated impacts of climate change in West Africa. Through the application of an innovative interactive board game, the authors explore gender-specific responses to climate variability in northwestern Benin and show that although women are equally aware of climate variability and share similar coping strategies with men, there are distinct gendered land use strategies, preferences and motivations. Interestingly, the paper demonstrates that traditional religious and spiritual norms and customs are also gendered. Likewise they show that gaming exercises provide a venue for women to share and negotiate changes in agricultural land use decisions such as crop cultivation area and choice of crop varieties. Finally, authors point out that such negotiation among and across gender roles is a key institutional feature which would possibly lead to dissimilar levels of vulnerability and coping strategies to increased climate variability. Hence, they stress the need to better understand the determinants of anticipatory learning (as opposed to a more reactive adaptive learning) as a critical component for building resilience at the agrarian landscape level.

Cohen et al. (2016) apply a qualitative study in the Solomon Islands, in the southwestern Pacific Ocean. They examine how socio-institutional factors, particularly social and gender norms, shape capacity to adapt and to innovate in order to provide insights for development interventions to enhance well-being in rural and coastal social–ecological systems. They report differences in beliefs about women–men hierarchies. Such beliefs are institutionalized through norms and customary ways of gender relations which do affect flexibility in terms of moving up or down the well-being ‘ladder’, livelihood mobility and physical mobility, which in turn shape capacity to adapt and to innovate. Such informal institutions are embedded within formal ones, such as village committees associated with external research programmes and organizations, including church groups and NGOs. Within such institutional context, authors find a barrier for women for agricultural innovation. The paper argues that social beliefs and expectations, which are often translated into social norms, are gendered, and can legitimize or hinder innovative actions, thus affecting adaptive capacity and willingness to invest in resilience building.

### Everyday practices and embodied experiences of adaptation

The need for a more grounded and localized understanding of GEC that recognizes the experiences of individuals and communities bound in local places and the cultural and symbolic impact of GEC has been increasingly demanded (Adger et al. 2009; Brace and Geoghegan 2011). This approach entails understanding how people make sense of GEC and how they cope with it in the context of their daily lives in which actions and responsibilities are negotiated and enacted under highly uneven power relations (Bee et al. 2015). In this context, understandings of gender become crucial because there are gender differences in the way people think, experience and adapt to GEC (MacGregor 2009). The concept of the everyday has been used by feminist researchers to explain how global processes and relations of power structure daily life in homes, neighbourhoods and communities (Dyck 2005; Bee et al. 2015). The everyday is the time–place where knowledge, action and experience come to matter and its study draws attention to issues of embodiment, difference and inequality in the lived experience of different subjects (Bee et al. 2015). Additionally, as remarked by Dyck (2005), close attention to everyday life helps to keep women visible in rapidly changing world conditions, rather than shade their activities beyond dominant models. Four papers in this special issue illustrate such focus (Buchanan et al. 2016; Buechler 2016; Ravera et al. 2016; Wilmer and Fernández-Giménez 2016).

Buechler (2016) draws on feminist political ecology to show the dynamic strategies of smallholders in orchards and home gardens in Northwest Mexico to changes in water availability in the face of climate change and neoliberal policies. She specifically highlights everyday spaces like home gardens, inhabited predominately by women, as sites that are largely invisible but important for their climate change mitigation and adaptation potential. Through an analysis of these spaces developed since 2007, she also shows how relations of power, specifically gender and class, work to hamper these strategies. Her analysis touches different scales, from the home to community and regional levels.

Ravera et al. (2016) explore adaptation strategies to climate change among farmers drawing on a feminist intersectional approach in two regions of India. They find gender differences in the perception and adoption of strategies to cope and adapt to climate change impacts and other concomitant drivers. However, they also disentangle how different dimensions of identity such as caste, wealth and age intersect with gender shaping the interactions between farmers and ecosystems. This intersectional approach also highlights how categories are changing and renegotiated under new drivers of change.

Wilmer and Fernández-Giménez (2016) explore resilience as an embodied practice, documenting how ranching women maintain livelihoods that support ranching as a living and a way of life in the face of weather variability, shifting rural demographics and economic opportunities in Southwestern US. Conceptualizing gender as material, discursive and contradictory, they locate women’s material practices and cultural perceptions of gender in the context of rangeland management in the Southwestern US using life-history interviews with 19 women ranchers. Their approach reveals the everyday meaning and experiences of women’s lives and the gendered practices of cultural resilience that contribute to ranching social–ecological system resilience.

Buchanan et al. (2016) use a capitals approach to analyse adaptive capacity and community resilience of Sami communities involved in reindeer husbandry in Sweden in the face of climate change and complex economic and socio-political conditions such as large-scale resource extraction, industrialization and past colonization. The authors overcome narrow assumptions about which activities are considered reindeer husbandry and who undertakes them and they propose a framework to conceptualize reindeer husbandry as the business, the land-based practices and the practices and cultural traditions.

## Footnotes

1 This term was coined by Donna Haraway (1988) largely based on feminist studies and it refers to the knowledge specific to a particular situation. Situational or situated knowledge is often embedded in language, culture or traditions and contingent to the history, power relations and geography.

2 According to the unique study on the topic (Banerjee and Bell 2007), less than 4 % of all articles in the top five journals in environmental social sciences published between 1980 and 2005 had an explicit treatment and reference to gender relations. More recently, a similar pattern has been found in climate change research (MacGregor 2009). To date there are no more systematic studies on the topic.

3 Resilience and Development: Mobilizing for Transformation (http://www.resilience2014.org/).

## References

[CR1] Adger WN (2006). Vulnerability. Journal article. Global Environmental Change.

[CR2] Adger WN, Dessai S, Goulden M, Hulme M, Lorenzoni I, Nelson DR, Naess LO, Wolf J (2009). Are there social limits to adaptation to climate change?. Climatic Change.

[CR3] Agarwal B (1992). The gender and environment debate: Lessons from India. Feminist Studies.

[CR4] Agarwal B (2010). Gender and green governance. The political economy of women’s presence within and beyond community forestry.

[CR5] Aregu L, Darnhofer I, Tegegne A, Hoekstra D, Wurzinger M (2016). The impact of gender-blindness on social-ecological resilience: The case of a communal pasture in the highlands of Ethiopia. Ambio.

[CR6] Arora-Jonsson S (2011). Virtue and vulnerability: Discourses on women, gender and climate change. Global Environmental Change.

[CR7] Arora-Jonsson S (2014). Forty years of gender research and environmental policy: Where do we stand?. Women’s Studies International Forum.

[CR8] Banerjee D, Bell MM (2007). Ecogender: Locating gender in environmental social science. Society & Natural Resources.

[CR9] Bee BA, Rice J, Trauger A (2015). A feminist approach to climate change governance: Everyday and intimate politics. Geography Compass.

[CR10] Berkes F, Folke C, Colding J (1998). Linking social and ecological systems: Management practices and social mechanisms for building resilience.

[CR11] Bhavnani K-K, Foran J, Kurian PA, Munshi D (2003). Feminist futures: Re-imagining women, culture and development.

[CR12] Biermann, M., K. Hillmer-Pegram, C. N. Knapp, and R. E. Hum. 2015. Approaching a critical turn? A content analysis of the politics of resilience in key bodies of resilience literature. *Resilience: International Policies, Practices and Discourses*

[CR13] Biggs R, Schlüter M, Schoon ML (2015). Principles for building resilience. Sustaining ecosystem services in social-ecological systems.

[CR14] Bose P (2012). Forest Rights. The micro-politics of decentralisation and forest tenure reform in tribal India.

[CR15] Bousquet F, Botta A, Alinovi L, Barreteau O, Bossio D, Brown K, Caron P, D’Errico M (2016). Resilience and development: Mobilizing for transformation. Ecology and Society.

[CR16] Brace C, Geoghegan H (2011). Human geographies of climate change: Landscape, temporality, and lay knowledges. Progress in Human Geography.

[CR17] Brown K (2014). Global environmental change I: A social turn for resilience?. Progress in Human Geography.

[CR18] Buchanan A, Reed MG, Lidestav G (2016). What’s counted as a reindeer herder? Gender and the adaptive capacity of Sami reindeer herding communities in Sweden. Ambio.

[CR19] Buechler S (2016). Gendered vulnerabilities and grassroots adaptation initiatives in home gardens and small orchards in Northwest Mexico. Ambio.

[CR20] Buechler S, Hanson A-M (2015). A political ecology of women, water and global environmental change.

[CR21] Carr ER (2008). Men’s crops and women’s crops: The importance of gender to the understanding of agricultural and development outcomes in Ghana’s central region. World Development.

[CR22] Carr ER, Thompson MC (2014). Gender and climate change adaptation in Agrarian settings: Current thinking, new directions, and research frontiers. Geography Compass.

[CR23] Cohen PJ, Lawless S, Dyer M, Morgan M, Saeni S, Teioli H, Kantor P (2016). Understanding adaptive capacity and capacity to innovate in social–ecological systems: Applying a gender lens. Ambio.

[CR24] Corbera E, Calvet-Mir L, Hughes H, Paterson M (2015). Patterns of authorship in the IPCC Working Group III report. Nature Climate Change.

[CR25] Cote M, Nightingale AJ (2012). Resilience thinking meets social theory: Situating social change in socio-ecological systems (SES) research. Progress in Human Geography.

[CR26] Crenshaw K (1991). Mapping the margins: Intersectionality, identity politics, and violence against women of color. Stanford Law Review.

[CR27] Cretney R (2014). Resilience for whom? Emerging critical geographies of socio-ecological resilience. Geography Compass.

[CR28] Dah-gbeto AP, Villamor GB (2016). Gender-specific responses to climate variability in a semi-arid ecosystem in northern Benin. Ambio.

[CR29] Díaz-Reviriego I, Fernández-Llamazares Á, Salpeteur M, Howard PL, Reyes-García V (2016). Gendered medicinal plant knowledge contributions to adaptive capacity and health sovereignty in Amazonia. Ambio.

[CR31] Djoudi H, Brockhaus M (2011). Is adaptation to climate change gender neutral? Lessons from communities dependent on livestock and forests in northern Mali. International Forestry Review.

[CR32] Djoudi H, Locatelli B, Vaast C, Asher K, Brockhaus M, Basnett Sijapati B (2011). Beyond dichotomies: Gender and intersecting inequalities in climate change studies. Ambio.

[CR33] Dyck I (2005). Feminist geography, the “everyday”, and local-global relations: hidden spaces of place-making. Canadian Geographer/Le Géographe canadien.

[CR34] Ellen RF, Parker P, Bicker A (2000). Indigenous environmental knowledge and its transformations: Critical anthropological perspectives.

[CR35] Elmhirst R (2011). Introducing new feminist political ecologies. Geoforum.

[CR36] Engle NL (2011). Adaptive capacity and its assessment. Global Environmental Change.

[CR37] Fabinyi M, Evans L, Foale SJ (2014). Social-ecological systems, social diversity, and power: Insights from anthropology and political ecology. Ecology and Society.

[CR38] Fairhead J, Leach M (1996). Misreading the African landscape: Society and ecology in a Forest-Savanna Mosaic.

[CR39] Folke C (2006). Resilience: The emergence of a perspective for social-ecological systems analyses. Global Environmental Change.

[CR40] Folke C, Carpenter SR, Walker B, Scheffer M, Chapin T, Rockström J (2010). Resilience thinking: Integrating resilience, adaptability and transformability. Ecology and Society.

[CR41] Fortmann L (2008). Participatory research in conservation and rural livelihoods: Doing science together.

[CR42] Gaard G (2011). Ecofeminism revisited: Rejecting essentialism and re-placing species in a material feminist environmentalism. Feminist Formations.

[CR100] Haraway DJ (1988). Situated knowledges: The science question in feminism and the privilege of partial perspective. Feminist studies.

[CR43] Harris LM (2006). Irrigation, gender, and social geographies of the changing waterscapes of Southeastern Anatolia. Environment and Planning D: Society and Space.

[CR44] Hernández-Morcillo M, Hoberg J, Oteros-Rozas E, Plieninger T, Gómez-Baggethun E, Reyes-García V (2014). Traditional ecological knowledge in Europe: Status Quo and insights for the environmental policy agenda. Environment: Science and Policy for Sustainable Development.

[CR45] Kaijser A, Kronsell A (2014). Climate change through the lens of intersectionality. Environmental Politics.

[CR46] Leach M (2016). Gender equity and sustainable development.

[CR47] MacGregor S (2009). A stranger silence still: The need for feminist social research on climate change. Sociological Review.

[CR48] McLaughlin P, Dietz T (2008). Structure, agency and environment: Toward an integrated perspective on vulnerability. Global Environmental Change.

[CR49] Meinzen-Dick R, Kovarik C, Quisumbing AR (2014). Gender and sustainability. Annual Review of Environment and Resources.

[CR50] Mies M, Shiva V (1993). Ecofeminism.

[CR51] Miller F, Osbahr H, Boyd E, Thomalla F, Bharwani S, Ziervogel G, Walker B, Van Der Leeuw S (2010). Resilience and vulnerability: Complementary or conflicting concepts?. Ecology and Society.

[CR52] Montana J, Borie M (2016). IPBES and biodiversity expertise: Regional, gender, and disciplinary balance in the composition of the interim and 2015 multidisciplinary expert panel. Conservation Letters.

[CR53] Nelson V, Stathers T (2009). Resilience, power, culture, and climate: A case study from semi-arid Tanzania, and new research directions. Gender & Development.

[CR54] Nightingale A (2003). A feminist in the forest: Situated knowledge and mixing methods in natural resource management. ACME.

[CR55] Nightingale A (2006). The nature of gender: Work, gender, and environment. Environment and Planning D: Society and Space.

[CR56] Nightingale AJ (2011). Bounding difference: Intersectionality and the material production of gender, caste, class and environment in Nepal. Geoforum.

[CR57] Nightingale AJ (2016). Adaptive scholarship and situated knowledges? Hybrid methodologies and plural epistemologies in climate change adaptation research. Area.

[CR58] Nilsson M, Griggs D, Visbeck M (2016). Map the interactions between sustainable development goals. Nature.

[CR59] Nygren A (1999). Local knowledge in the environment-development discourse: From dichotomies to situated knowledges. Critique of Anthropology.

[CR60] O’Shaughnessy S, Krogman NT (2011). Gender as contradiction: From dichotomies to diversity in natural resource extraction. Journal of Rural Studies.

[CR61] Ogden L, Heynen N, Oslender U, West P, Kassam K-A, Robbins P (2013). Global assemblages, resilience, and Earth Stewardship in the Anthropocene. Frontiers in Ecology and the Environment.

[CR62] Pardo-de-Santayana M, Pieroni A, Puri RK, Pardo-de-Santayana M, Pieroni A, Puri RK (2010). The ethnobotany of Europe, past and present. The Ethnobotany in the New Europe: People, health and wild plant resources.

[CR63] Ravera, F., and D. Tarrasón, published in Bose and Savyasachi. (Eds.) 2014. Landscaping actually: Forests to farms through a gender lens. E-book for CIAT’s FTA integrating Gender, Cali. https://issuu.com/ciat-ftagender/docs/final_ebook.

[CR64] Ravera F, Martín-López B, Pascual U, Drucker A (2016). The diversity of gendered adaptation strategies to climate change of Indian farmers: A bottom-up feminist intersectional approach. Ambio.

[CR65] Reid P, Vogel C (2006). Living and responding to multiple stressors in South Africa—Glimpses from KwaZulu-Natal. Global Environmental Change.

[CR66] Resurreccion BP, Elmhirst R (2008). Gender and natural resource management: Livelihoods, mobility and interventions.

[CR67] Resurrección BP (2013). Persistent women and environment linkages in climate change and sustainable development agendas. Women’s Studies International Forum.

[CR68] Rocheleau D (1995). Maps, numbers, text, and context: Mixing methods in feminist political ecology. Professional Geographer.

[CR69] Rocheleau D, Edmunds D (1997). Women, men and trees: Gender, power and property in forest and agrarian landscapes. World Development.

[CR70] Rocheleau D, Thomas-Slayter B, Wangari E (1996). Feminist political ecology: Global issues and local experience.

[CR71] Rockström J, Steffen W, Noone K, Persson Å, Chapin FS, Lambin EF (2009). A safe operating space for humanity. Nature.

[CR72] Sandilands C (1999). The good-natured feminist. Ecofeminism and the quest for democracy.

[CR73] Seager J (2003). Rachel Carson died of breast cancer: The coming of age of feminist environmentalism. Signs.

[CR74] Segnestam L (2009). Division of capitals—What role does it play for gender-differentiated vulnerability to drought in Nicaragua?. Community Development.

[CR75] Shiva V (1988). Staying alive: Women, ecology, and development.

[CR76] Smucker TA, Wangui EE (2016). Gendered knowledge and adaptive practices: Differentiation and change in Mwanga District, Tanzania. Ambio.

[CR77] Thompson-Hall, M., E.R. Carr, and U. Pascual. 2016. Enhancing and expanding intersectional research for climate change adaptation in agrarian settings. *Ambio*. doi:10.1007/s13280-016-0827-0. 10.1007/s13280-016-0827-0 PMC5120020 27878538

[CR78] Tschakert P (2012). From impacts to embodied experiences: Tracing political ecology in climate change research. Geografisk Tidsskrift-Danish Journal of Geography.

[CR79] Tschakert P, Dietrich KA (2010). Anticipatory learning for climate change adaptation and resilience. Ecology and Society.

[CR80] Tschakert P, Machado M (2012). Gender justice and rights in climate change adaptation: Opportunities and pitfalls. Ethics and Social Welfare.

[CR81] Tschakert P, Tuana N, Dugard J, St Clair AL, Gloppen S (2013). Situated resilience: Reframing vulnerability and security in the context of climate change. Climate talk: Rights, poverty and justice.

[CR82] Turner BL (2010). Vulnerability and resilience: Coalescing or paralleling approaches for sustainability science?. Global Environmental Change.

[CR83] Turner DM (2013). Political ecology I: An alliance with resilience?. Progress in Human Geography.

[CR84] UN United Nations. 2012. *The future we want: Outcome document adopted at Rio*+*20.*

[CR85] UN Women. 2014. *World survey on the role of women in development 2014. Gender equality and sustainable development*. New York

[CR86] UNCED. 1992. The Rio declaration on environment and development. In *Agenda 21*, 366–368

[CR30] van Dijk, H., and P. Bose. 2016. Dryland landscapes: Forest management, gender and social diversity in Asia and Africa. In *Dryland forests. Management and social diversity in Africa and Asia*, 3–21. New York: Springer.

[CR87] Verma R, Molden D, Hurni H, Zimmermann AB, von Dach SW (2014). Special issue: Gender and sustainable development in mountains—Transformative innovations, tenacious resistances. Mountain Research and Development.

[CR88] Walker B, Holling CS, Carpenter SR, Kinzig A (2004). Resilience, adaptability and transformability in social-ecological systems. Journal Article. Ecology and Society.

[CR89] Wilmer H, Fernández-Giménez ME (2016). Some years you live like a coyote: Gendered practices of cultural resilience in working rangeland landscapes. Ambio.

